# Treatment patterns, unmet need, and impact on patient-reported outcomes of psoriatic arthritis in the United States and Europe

**DOI:** 10.1007/s00296-018-4195-x

**Published:** 2018-11-13

**Authors:** Alice Gottlieb, Jordi Gratacos, Ara Dikranian, Astrid van Tubergen, Lara Fallon, Birol Emir, Laraine Aikman, Timothy Smith, Linda Chen

**Affiliations:** 1 Department of Dermatology, New York Medical College at Metropolitan Hospital, 1901 First Avenue, Floor 14B, New York, NY 10021 USA; 2 Department of Rheumatology, University Hospìtal Parc Taulí Sabadell, Barcelona, Spain; 3 Cabrillo Center for Rheumatic Disease, San Diego, CA USA; 4 Department of Medicine, Division of Rheumatology, Maastricht University Medical Center, Maastricht, Netherlands; 5 Pfizer Inc, Montreal, QC Canada; 6 Pfizer Inc, New York, NY USA; 7 Pfizer Ltd, Sandwich, UK

**Keywords:** Arthritis, psoriatic, Health status, Patient-reported outcome measures, Surveys and questionnaires, Therapeutics

## Abstract

Psoriatic arthritis (PsA) is a chronic, inflammatory disease. The effects of PsA real-world treatment patterns on patient-reported outcomes in the US and 5 European countries (EU5; France, Germany, Italy, Spain, UK) were evaluated. Respondents from the 2016 National Health and Wellness Survey received advanced therapies (e.g., biologic disease-modifying antirheumatic drugs [DMARDs]), other therapies, (e.g., conventional synthetic DMARDs), or no treatment. Assessments included demographics, disease severity (patient-reported), comorbidities (Charlson Comorbidity Index), health status (Short Form-36 Health Survey), depression (Patient Health Questionnaire-9), work productivity (Work Productivity and Activity Index), and treatment adherence (Morisky Medication Adherence Scale-8). Overall, 1037 respondents from the US and 947 respondents from the EU5 were included. Of these, 21.7% US and 7.3% EU5 respondents received advanced therapies; 16.6% and 28.5%, other therapies; and 61.7% and 64.2%, no treatment, respectively. During treatment with advanced or other therapies, 40.8–54.7% US and 57.7–58.9% EU5 respondents self-reported moderate or severe PsA. Respondents receiving advanced therapies had the highest Charlson Comorbidity Index score (US, 1.25; EU5, 1.42); the lowest scores were with no treatment (0.52 and 0.49, respectively). Employment was lowest with other therapies (US, 47.7%; EU5, 41.1%). Overall work impairment was reported by 57.9% US and 62.6% EU5 respondents receiving advanced therapies. Medication adherence was generally low in the US and medium in the EU5 (Morisky Medication Adherence Scale-8: low, US 40.1–46.7%, EU5, 29.0–35.2%; medium, US 29.3–36.1%, EU5 37.8–49.3%; high, US 23.8–24.0%; EU5, 21.7–27.0%). Advanced and other therapies reduced PsA severity; however, > 40% of respondents reported moderate or severe PsA during treatment. Better management and adherence may reduce unmet need and disease burden. Further work is required to improve PsA diagnosis and time to treatment initiation.

**Electronic supplementary material:**

The online version of this article (10.1007/s00296-018-4195-x) contains supplementary material, which is available to authorized users.

## Introduction

Psoriatic arthritis (PsA) is a chronic, debilitating, inflammatory disease. The domains of PsA have been defined by the Group for Research and Assessment of Psoriasis and Psoriatic Arthritis (GRAPPA) as peripheral arthritis, axial disease, enthesitis, dactylitis, psoriasis (PsO), and nail disease [1]. More recently, the GRAPPA and Outcome Measures in Rheumatology (OMERACT) working group defined a core domain set including pain, Patient’s Global Assessment of disease activity, physical function, health-related quality of life (HRQoL), fatigue, and systemic inflammation, in addition to musculoskeletal and skin disease [2, 3], reflecting the growing significance of patient-reported outcomes (PROs) for monitoring disease progression and treatment response. Indeed, in the Multinational Assessment of Psoriasis and Psoriatic Arthritis (MAPP) population-based survey, 88% of patients with PsA reported current joint pain or soreness, and 53% rated their disease as severe [4].

Due to the complexity and range of symptoms involved, PsA may be diagnosed and managed by both dermatologists and rheumatologists. However, as PsA is often misdiagnosed or undiagnosed [5, 6], estimates of its prevalence vary widely (ranging from 0.2 to 1.0% in the US [7, 8] and from 0.1 to 2.0% in Europe [9–13]) and is likely higher than reported [14]. In addition, the proportion of patients suffering from both PsO and PsA has been estimated to range from 6 to 42% [5, 6, 14–17].

PsA is associated with considerable disease burden, increased healthcare costs, and impairments in HRQoL and work productivity [4, 18–20], and is associated with substantial comorbidities and extra-articular manifestations.

PsO and rheumatoid arthritis (RA), unique conditions that share symptoms with PsA, are associated with a number of comorbidities including increased risk of cardiovascular events and disease, mortality, infections, and malignancies compared with the general population [21–29]. Patients with PsO also have higher levels of anxiety and depression, with the risk of these comorbidities further increased when suffering from both PsO and PsA [30, 31].

Treatment guidelines from GRAPPA [1], the American Academy of Dermatology (AAD) [32], and the European League Against Rheumatism (EULAR) [33] specify that treatment for PsA should reflect disease characteristics and response to prior treatment. Indeed, treatment should be initiated upon diagnosis by a dermatologist or rheumatologist with the goals of alleviating signs and symptoms, improving functional status, inhibiting structural damage, and improving HRQoL parameters [1, 32, 33]. Furthermore, the treatment target should be remission or, where appropriate, minimal/low disease, with treatment decisions reflecting shared decision-making between patient and physician considering all attributes of the disease and treatment [1, 32, 33].

EULAR guidelines recommend that patients with active PsA receive non-steroidal anti-inflammatory drugs (NSAIDs), then conventional synthetic disease-modifying antirheumatic drugs (csDMARDs) if they do not respond to NSAIDs, followed by a biologic DMARD (usually starting with a tumor necrosis factor inhibitor [TNFi]) or targeted synthetic DMARD (such as the phosphodiesterase 4-inhibitor [PDE-4i], apremilast) if needed [33]. AAD guidelines recommend methotrexate, TNFi, or a combination of both as first-line treatment in moderate or severe PsA [32]. Non-pharmacologic strategies, such as patient education, exercise, and weight reduction, may also be used to manage the disease [33].

Despite the development of treatment guidelines by GRAPPA, AAD, and EULAR, along with a greater understanding of the disease burden of PsA, the effects of real-world treatment patterns on PROs and health status have not been fully evaluated to date. Here, we report a descriptive, exploratory analysis of data from the cross-sectional, 2016 National Health and Wellness Survey (NHWS). The objectives of this analysis were to characterize patients with self-reported PsA and to describe the effect of treatment patterns (current treatment, or lack of treatment) on PROs, with the aim of improving understanding of how treatment affects health status in the real world in the US and EU5.

## Methods

### Participants

Data were taken from the 2016 NHWS [34], a self-administered, web-based, participant-completed questionnaire designed to provide a representative sample of adults (aged ≥ 18 years) from the US and five European countries (the EU5: France, Germany, Italy, Spain, and the UK) through a randomized sampling framework. Potential respondents were identified through the general panel of Lightspeed Research and were recruited through opt-in email, coregistration with Lightspeed Research partners, e-newsletter campaigns, banner placements, and both an internal and external affiliate network. All panel members explicitly agreed to become part of the panel and receive invitations to participate in online surveys. Participants provided an in-depth demographic profile at registration and completed the survey knowing that any identifying information and individual answers would be kept confidential.

### Variables

NHWS respondents were asked ‘Which of the following conditions have you ever experienced?’ and were provided with a list of conditions, with PsA listed under ‘chronic pain conditions’. Respondents who reported having PsA were directed to complete a section called the “arthritis module”. Respondents who did not self-report having PsA or who self-reported having PsA, but did not complete the arthritis module were excluded from the analysis. Respondents reporting they had PsA who completed the arthritis module were grouped by mutually exclusive categories based on self-reports of treatments currently being used to treat their disease. Three pharmacologic treatment categories were defined: advanced therapies (including TNFi, interleukin (IL)-12/23 and IL-17 antagonists, and PDE-4i; patients were included in this group regardless of any non-advanced therapies currently being used), treatment with other therapies (including participants reporting an absence of advanced therapy and utilization of any csDMARDs, cyclooxygenase 2 inhibitors, NSAIDs, glucocorticoids, and topical medications), and no treatment.

PRO data were collected as components within the NHWS questionnaire and summarized descriptively. PRO instruments included the Short Form-36 Health Survey (SF-36) to measure health status, including physical health (Physical Component Summary [PCS]) and mental health (Mental Component Summary [MCS]) [35]; the Work Productivity and Activity Index (WPAI), including domains to measure ability to work and perform regular daily activities [36]; the Patient Health Questionnaire (PHQ)-9, to measure the severity of depression [37]; and the Morisky Medication Adherence Scale to measure adherence to treatment [38]. Patient-reported use of healthcare resources within the previous 6 months was also recorded.

Patients were asked to self-report the current and pre-treatment severity of their PsA as mild, moderate, or severe, based on their perception of their current health state and recollection of their health state prior to receiving treatment; no specific definitions or criteria were provided to patients for self-rating of PsA severity. Sociodemographics and health history details were also collected.

### Statistical methods

Descriptive statistics were calculated for personal characteristics and included means (± standard deviation) and relative frequencies (%) as applicable. Basic inferential statistical tests such as Chi-square (for categorical data) and *t* tests (for continuous data) were used to assess unadjusted associations. All analyses were performed using SAS version 14.1. No missing value imputation was performed. No multiplicity correction adjustments were made.

## Results

### Respondents

In total, 97,503 US and 80,600 EU5 adults completed the 2016 NHWS; 1140 (1.17%) respondents in the US and 1085 (1.35%) in the EU5 self-reported having PsA.

Among US respondents, 1037 who reported having PsA completed the arthritis module and provided information on treatment. Of these, 225 (21.7%) reported receiving advanced therapies, 172 (16.6%) other therapies, and 640 (61.7%) no treatment. In the EU5, 947 respondents who reported having PsA completed the arthritis module and provided information on treatment, 69 (7.3%) reported receiving advanced therapies, 270 (28.5%) other therapies, and 608 (64.2%) no treatment.

Age distribution was similar across US and EU5 patients (Table 1). Significant differences were observed between patients receiving advanced therapies and other therapies, and between patients receiving other therapies and no treatment, for both US and EU5 patients. The proportion of female patients was similar in the advanced therapies and no treatment groups (US, 53.3% and 48.9%; EU5, 52.2% and 51.2%, respectively), but a greater proportion of female respondents received other therapies group in both the US (61.1%, *p* < 0.01) and EU5 (64.1%) compared with the no treatment group (*p* < 0.001; Table 1). Differences in body mass index (BMI) and smoking history can be found in Table 1 for the US and EU5, along with a breakdown of individual European countries in the EU5 in Online Resource 1. In both the US and EU5, patients receiving advanced therapies had significantly higher age-adjusted Charlson Comorbidity Index score (1.25 and 1.42, respectively), vs. those receiving no treatment (0.52, *p* < 0.001 and 0.49, *p* ≤ 0.05, respectively; Table 1). Significant differences were also observed between other therapies (US 0.96, EU5 0.80) and no treatment (both *p* < 0.001).

**Table 1:** Demographics and characteristics of survey respondents who reported having PsA

	US patients			EU5 patients		
	Advanced therapies *N* = 225	Other therapies *N* = 172	No treatment *N* = 640	Advanced therapies *N* = 69	Other therapies *N* = 270	No treatment *N* = 608
Age in years, mean (SD)	47.7 (14.4)***	53.6 (14.5)^†††^	46.6 (17.0)	50.5 (13.1)^‡‡‡^	56.5 (13.4)^§§§^	49.7 (16.4)
Female, *n* (%)	120 (53.3)	105 (61.1)^††^	313 (48.9)	36 (52.2)	173 (64.1)^§§§^	311 (51.2)
White ethnicity, *n* (%)	191 (84.9)^††^	146 (84.9)^††^	479 (74.8)	NR	NR	NR
Employed,^a^*n* (%)	159 (70.7)	82 (47.7)	356 (55.6)	38 (55.1)	111 (41.1)	352 (57.9)
Employed full time^b^	135 (84.9)***^†††^	57 (69.5)	252 (70.8)	24 (63.2)	65 (58.6)^§§§^	227 (64.5)
Employed part-time^b^	8 (5.0)*^††^	14 (17.1)	60 (16.9)	10 (26.3)	27 (24.3)	76 (21.6)
Self-employed^b^	16 (10.1)	11 (13.4)	44 (12.4)	4 (10.5)	19 (17.1)	49 (13.9)
BMI kg/m^2^, *n* (%)						
*N*	211	167	605	63	253	561
< 18.5	12 (5.7)	4 (2.4)	34 (5.6)	0 (0.0)	7 (2.8)	23 (4.1)
18.5–< 25	63 (29.9)	45 (27.0)	163 (26.9)	21 (33.3)	72 (28.5)	197 (35.1)
25–< 30	55 (26.1)	36 (21.6)	178 (29.4)	16 (25.4)	88 (34.8)	196 (34.9)
≥ 30	81 (38.4)	82 (49.1)	230 (38.0)	26 (41.3)	86 (34.0)	145 (25.9)
Current smoker, *n* (%)	78 (34.7)	48 (27.9)	184 (28.8)	33 (47.8)^‡‡§§^	82 (30.4)	195 (32.1)
Adjusted Charlson Comorbidity Index score, mean (SD)^c^	1.25 (3.16)^†††^	0.96 (1.39)^†††^	0.52 (1.12)	1.42 (3.17)^§^	0.80 (1.22)^§§§^	0.49 (1.10)

*BMI* body mass index; *EU5* France, Germany, Italy, Spain, UK; *NR* not recorded, *PsA* psoriatic arthritis, *SD* standard deviation

*p < 0.05, ***p < 0.001 vs. other therapies within the US; ^††^*p* < 0.01, ^†††^*p* < 0.001 vs. no treatment within the US; ^‡‡^*p* < 0.01, ^‡‡‡^*p* < 0.001 vs. other therapies within the EU5; ^§^*p* ≤ 0.05, ^§§^*p* < 0.01, ^§§§^*p* < 0.001 vs. no treatment within the EU5

^a^Full- or part-time employment or self-employed

^b^Calculated as a proportion of total employed

^c^Higher scores represent greater comorbidity

In the US and EU5, 57.6% and 52.9% of patients, respectively, were employed and the majority of these (US, 74.4%; EU5, 63.1%) were employed full time. In both the US and EU5, the treatment group receiving other therapies had the lowest proportion of employed patients (47.7% and 41.1%, respectively; Table 1). The proportion of full-time employed US respondents that received advanced therapies (84.9%) was higher vs. those reporting other therapies (69.5%) or no treatment (70.8%). Among self-employed or part-time employed US patients, few reported receiving advanced therapies (10.1% and 5%, respectively). In contrast, the distribution of EU5 patients was similar across all three treatment groups for those in full-time employment (advanced therapies, 63.2%; other therapies, 58.6%; and no treatment, 64.5%) and few EU5 patients who were self-employed (10.5%) reported receiving advanced therapies, while 26.3% of patients employed part-time reported receiving advanced therapy. Details of the demographics and characteristics of the individual countries in the EU5 can be seen in Online Resource 1.

In the US, the no treatment group had the lowest proportion of patients with some form of health insurance (86.4%) vs. advanced (93.3%) and other therapies groups (95.4%; both *p* < 0.01 vs. no treatment); Medicare and coverage through a current or former employer were the most common sources of insurance (Online Resource 2). In the EU, 92.0% of patients had health insurance, with public insurance alone being the most common (Online Resource 3). A similar proportion of patients had health insurance across the treatment groups in EU5, with significant differences between advanced therapies and other therapies (*p* < 0.01) and between other therapies and no treatment (*p* < 0.05) in the UK only.

### Self-reported PsA severity

**Fig. 1:**
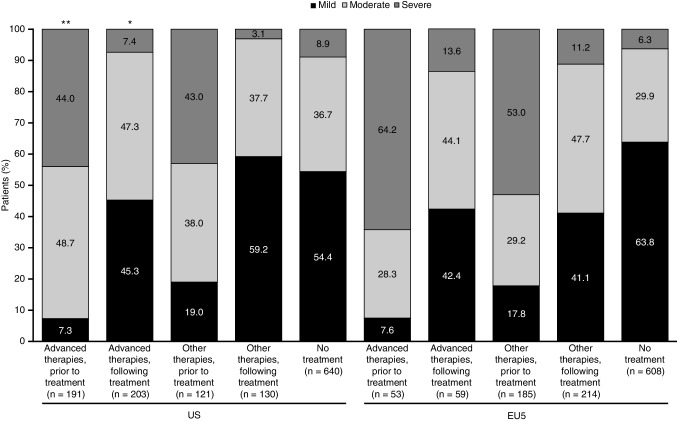
Severity of PsA prior to and when receiving treatment based on patient self-report in US and EU5 patients. *p < 0.05; **p < 0.01 for patients with moderate-to-severe disease receiving advance therapies vs. other therapies within the respective region. *EU5* France, Germany, Italy, Spain, UK; *PsA* psoriatic arthritis

Prior to treatment with advanced or other therapies, 81.0–92.7% and 82.2–92.5% of respondents in the US and EU5, respectively, self-reported moderate or severe PsA. In the US, fewer patients reported severe disease following treatment with advanced (7.4%) or other therapies (3.1%) compared with pre-treatment (44.0% and 43.0%, respectively), although 54.7% of patients receiving advanced therapies and 40.8% of patients receiving other therapies still rated their PsA as moderate or severe (Fig. 1). Similar changes were seen in the EU, with reductions in patient-reported severe disease from 64.2 to 13.6% for those reporting use of advanced therapies and 53.0–11.2% for other therapies for pre-treatment vs. following treatment, respectively. Again, 57.7% and 58.9% of patients still rated their PsA as moderate or severe for advanced and other therapies, respectively (Fig. 1).

Among patients who self-reported receiving no treatment, in both the US and the EU5, the highest proportion of patients self-reported mild disease (US, 54.4%; EU5, 63.8%), and the lowest proportion of patients self-reported severe disease (US, 8.9%; EU5, 6.3%; Fig. 1) compared with the advanced and other treatment groups prior to treatment. Details of the self-reported PsA severity of the individual countries in the EU5 can be seen in Online Resource 4.

### Patient-reported outcomes

SF-36 MCS and PCS scores, and PHQ-9 scores, were broadly similar in the US and EU5 (Table 2) [39]. Differences between treatments in SF-36 PCS scores were statistically significant for advanced therapies and other therapies compared with no treatment for both US (*p* < 0.01 and *p* < 0.001, respectively) and EU5 patients (*p* < 0.001 for both).

**Table 2:** Post-treatment outcome scores by treatment type

	US patients			EU5 patients		
	Advanced therapies *N* = 225	Other therapies *N* = 172	No treatment *N* = 640	Advanced therapies *N* = 69	Other therapies *N* = 270	No treatment *N* = 608
SF-36 PCS score, mean (SD)	39.9 (9.3)^††^	38.3 (10.9)^†††^	42.2 (9.8)	35.8 (9.7)^§§§^	37.7 (9.7)^§§§^	43.9 (8.4)
SF-36 MCS score, mean (SD)	41.5 (11.7)	42.1 (11.0)	40.7 (11.0)	37.8 (10.7)	40.2 (11.7)	39.8 (10.5)
WPAI domain scores, mean (SD)^a^						
Absenteeism (work time missed), %	*N* = 150 20.8 (22.7)	*N* = 79 19.3 (26.8)	*N* = 339 22.4 (26.8)	*N* = 36 25.9 (28.9)	*N* = 110 28.1 (35.8)	*N* = 338 20.8 (27.8)
Presenteeism (impairment at work), %	*N* = 153 53.5 (30.1)*	*N* = 76 45.1 (29.8)	*N* = 339 48.8 (31.4)	*N* = 36 57.2 (30.2)^‡‡§^	*N* = 96 41.9 (29.6)	*N* = 328 44.5 (29.3)
Overall work impairment, %	*N* = 149 57.9 (32.4)	*N* = 76 49.9 (33.1)	*N* = 333 55.0 (33.5)	*N* = 36 62.6 (33.0)^‡§^	*N* = 96 48.1 (33.3)	*N* = 328 51.1 (32.6)
Activity impairment, %	*N* = 225 56.4 (28.2)^†^	*N* = 172 55.3 (27.7)	*N* = 640 51.0 (28.9)	*N* = 69 62.6 (25.5)^§§§^	*N* = 270 57.6 (26.6)^§§§^	*N* = 608 48.3 (28.9)
Healthcare resource use in past 6 months, mean number (SD)						
Visits to ER	1.1 (2.5)*	0.7 (1.5)	0.9 (2.5)	1.0 (1.8)^‡‡^	0.5 (1.6)	0.6 (2.3)
Hospitalizations	1.1 (3.5)^††^	0.4 (1.1)	0.7 (2.8)	0.9 (1.6)^§§^	0.5 (2.4)	0.4 (1.1)
HCP visits	7.5 (8.6)^†^	8.1 (10.7)^†^	5.9 (10.4)	16.0 (20.8)^‡§§§^	10.7 (12.8)^§§§^	6.7 (7.8)
Visits to rheumatologist	0.5 (0.5)***^†††^	0.3 (0.4)^†††^	0.1 (0.2)	0.6 (0.5)^‡‡§§§^	0.4 (0.5)^§§§^	0.1 (0.3)
Visits to dermatologist	0.3 (0.5)*^†††^	0.2 (0.4)	0.2 (0.4)	0.4 (0.5)^‡§§^	0.3 (0.5)^§^	0.2 (0.4)
PHQ-9 total score, mean (SD)	*N* = 35 8.9 (7.8)	*N* = 45 7.0 (6.7)	*N* = 138 9.6 (8.0)	*N* = 22 7.7 (7.0)	*N* = 45 9.1 (6.8)	*N* = 137 7.8 (7.1)
MMAS-8, *n* (%)						
Low (< 6)	105 (46.7)	69 (40.1)	NA	20 (29.0)	95 (35.2)	NA
Medium (6–< 8)	66 (29.3)	62 (36.1)	NA	34 (49.3)	102 (37.8)	NA
High (8)	54 (24.0)	41 (23.8)	NA	15 (21.7)	73 (27.0)	NA

*ER* emergency room; *EU5* France, Germany, Italy, Spain, UK; *HCP* healthcare professional; *MCS* Mental Component Summary; *MMAS* Morisky Medication Adherence Scale; *NA* not applicable; *PCS* Physical Component Summary; *PHQ* Patient Health Questionnaire; *SD* standard deviation; *SF-36* Short Form-36 health survey; *WPAI* Work Productivity and Activity Index

*p < 0.05, ***p < 0.001 vs. other therapies within the US; ^††^*p* < 0.01, ^†††^*p* < 0.001 vs. no treatment within the US; ^‡‡^*p* < 0.01, ^‡‡‡^*p* < 0.001 vs. other therapies within the EU5; ^§^*p* ≤ 0.05, ^§§^*p* < 0.01, ^§§§^*P* < 0.001 vs. no treatment within the EU5

^a^The WPAI yields four types of scores: (1) Absenteeism (work time missed); (2) Presenteeism (impairment at work/reduced on-the-job effectiveness); (3) Work productivity loss (overall work impairment/absenteeism plus presenteeism); (4) Activity impairment [39]

As measured by their completion of the WPAI, patients in both the US and the EU5 reported missing work. The range of absenteeism was similar for both US (19.3–22.4% total work time) and EU5 (20.8–28.1% total work time) across all treatment groups (Table 2). The percentage of patients with presenteeism was highest for patients receiving advanced therapies (US, 53.5% [*p* < 0.05 vs. other therapies]; EU5, 57.2% [*p* < 0.01 vs. other therapies; *p* < 0.05 vs. no treatment]), followed by those with no treatment (US, 48.8%; EU5, 44.5%) and those receiving other therapies (US, 45.1%, *p* < 0.05 vs. advanced therapies; EU5, 41.9%, *p* < 0.01 vs. advanced therapies; Table 2). The largest proportion of patients with overall work productivity loss in both regions was reported by those receiving advanced therapies, with significant differences between advanced therapies (62.6%) and other therapies (48.1%) and no treatment (51.1%, both *p* < 0.05) in EU5 patients.

Patients in the advanced therapies group reported significantly higher levels of healthcare resource use of all types than other therapies and no treatment during the past 6 months in both the US and EU5 (Table 2). This included visits to the emergency room, hospitalizations, visits to a rheumatologist, and visits to a dermatologist. The exception was healthcare professional (HCP) visits which was highest in the US for patients receiving other therapies and for patients receiving advanced therapies in EU5 (Table 2).

In the US, patients in both the advanced therapies and other therapies groups were more likely to self-report low adherence (Morisky Medication Adherence Scale [MMAS]-8, < 6; 46.7% and 40.1%, respectively). In the EU5, patients in both the advanced and other therapies groups were more likely to self-report medium adherence (MMAS-8, 6 to <8; 49.3% and 37.8%; Table 2).

## Discussion

In this cross-sectional, descriptive, exploratory analysis of 2016 NHWS PRO survey data, 1.17% of survey respondents in the US and 1.35% of respondents in the EU5 self-reported having PsA. This is higher than the mean reported adult population prevalence estimates of 0.19% in the UK [12] and 1% in the US [8]; however, estimates are variable and the estimated prevalence in this study is consistent with the upper ranges found in the literature. In addition, this difference may be a reflection of respondents who consider themselves to have PsA, but might actually have some form of musculoskeletal disorder alongside PsO or skin manifestation.

These data provide insight into current treatment patterns in the US and EU5, reporting that 62% and 64% of patients with PsA, respectively, self-reported receiving no treatment. However, as previously noted, as PsA was self-reported and no clinical diagnosis was given in this study, it is possible that patients self-reporting PsA may have suffered from PsO or a skin manifestation alongside a musculoskeletal disorder and these participants may have been disproportionally represented in the no treatment group. Despite this, the results were consistent with the findings of another study of PsA impact and unmet treatment needs in North America and Europe, in which 58% of patients self-reporting PsA when responding to a telephone survey reported receiving no treatment or topical therapy only [4, 9]. In this analysis, the majority of patients in the US and EU5 receiving no treatment considered themselves to have mild or moderate disease (91.1 and 93.8%, respectively) and had the lowest Charlson Comorbidity index score amongst the treatment groups.

Prior to treatment, the majority of patients reported moderate or severe PsA (US: 81.0–92.7%, EU5: 82.2–92.5%). For patients receiving treatment with either advanced or other therapies, fewer patients self-reported severe disease (US: 7.4 and 3.1%; EU5: 13.6 and 11.2%, respectively), and a higher proportion reported mild disease (US: 45.3 and 59.2%, respectively; EU5: 42.4 and 41.1%, respectively), compared with self-reported pre-treatment severity, suggesting that patients responded to treatment. Despite this improvement, 54.7 and 40.8% of patients in the US and 57.6 and 58.9% of patients in the EU5 still rated their PsA as moderate or severe for advanced and other therapies, respectively.

This was a population-based survey relying on self-reported PsA rather than a study conducted in rheumatology or dermatology clinics. This likely impacted the patient population identified, resulting in a larger number of patients with lower disease activity, and therefore, a lower proportion of patients receiving advanced therapies and a higher proportion of patients receiving no treatment than might be expected. Despite this, both age distribution and the proportions of female/male patients were similar across the US and EU5 treatment groups. The largest proportion of patients in the US with a high BMI received other therapies, whereas in the EU5, the largest proportion of patients with a high BMI received advanced therapies. In addition, in both the US and EU5, patients receiving advanced therapies had the highest age-adjusted Charlson Comorbidity Index score compared with other treatment groups. These data indicate that high BMI and comorbidities are common in PsA, with the highest disease burden being observed in patients receiving advanced therapies.

The findings of these analyses confirmed the impact of PsA on HRQoL, with SF-36 PCS and MCS scores below the normative values seen in the general population [40]. SF-36 PCS scores were similar to those reported in a literature review of studies of patients with PsA, while SF-36 MCS scores were low compared with those reported in the same review [41]. The negative impact on the physical dimension of HRQoL is not unexpected considering the pain and swelling of joints often experienced by patients with PsA. Low SF-36 MCS scores likely reflect the well-documented impact of PsO and RA on HRQoL [42–46]; many patients experience psychosocial problems and emotional distress due to the unsightly appearance of the skin lesions [43].

The majority of US patients were in full-time employment and a greater proportion of these patients received advanced therapies compared with other treatment groups. In EU5, full-time employment levels were similar across treatment groups. Similar to the previous investigations in PsA [4, 47], productivity was reported to have been impaired in patients self-reporting PsA in this survey. These findings are consistent with those in an NHWS 2010–2013 population-based survey in the same five European countries as the current study, in which overall work impairment was 52% [47]. Similarly, in the MAPP population-based survey, 32% of patients with PsA reported missing work in the previous 12 months because of their PsA, and 32% reported that PsA had impacted their ability to work full time, although the extent of the work missed was not quantified [4]. Work missed by patients with PsA in an international clinical trial of certolizumab pegol was lower than in the current study, ranging from 5 to 9% across treatment groups at baseline [48]. Finally, activity impairment was slightly higher in patients receiving advanced therapies than those receiving other therapies or no treatment; this could be related to the higher disease severity reported by these patients.

Self-reported medication adherence was generally poor in the current study. This may be a reflection of access to advanced therapies in the EU5, with public insurance coverage being more prominent compared with the US healthcare systems. A recent systematic review of medication adherence in patients with a range of conditions, including PsA, identified varying levels of non-adherence, which can impact negatively on health outcomes and have associated economic costs [49]. In the systematic review, psychosocial factors, such as perceived treatment efficacy and safety, emotional well-being, HCP–patient relationship, and practical barriers, were shown to be key factors in determining patient adherence to treatment, while demographic and clinical factors showed less of an association with adherence [49]. Since reason for non-adherence was not explored in the current study, it is possible that some of the factors identified by Vangeli et al. influenced patients’ decisions regarding medication adherence [49]. It is also possible that in the US, insurance coverage for advanced therapies is likely a negative factor impacting adherence.

A number of limitations of this analysis must be acknowledged. Patients were included if they selected PsA in response to the question ‘Which of the following conditions have you ever experienced?’, where PsA was listed under ‘chronic pain conditions’. The diagnosis of PsA was not further validated by physician or chart review. Grouping of patients by treatment was based on self-reports of treatments used to treat arthritis; it is, therefore, possible that some patients might have been receiving treatment for PsO that might also be effective for PsA, but would not necessarily have been captured. Disease severity (mild, moderate, or severe) was not defined and was determined based on a patient’s response to the question ‘What is the level of severity of your condition?’, with no criteria provided to guide self-rating of severity of PsA. Results may, therefore, differ from those that would have been obtained if respondents had been required to receive a formal physician-reported PsA diagnosis and severity rating. It is worth noting, however, that participants receiving advanced therapies and other therapies reported seeing a rheumatologist or dermatologist more frequently than those participants receiving no treatment, lending support to the self-reported PsA in these treatment groups. Patients were not required to be under the care of a clinician for their PsA, which might have resulted in different treatment patterns, with fewer patients being treated, particularly with advanced therapies, compared with patients under the care of a rheumatologist or dermatologist. Pre-treatment severity levels were based on patients’ recollection of their health state before they started treatment. Information regarding the delay between a patient starting their current treatment and responding to the survey was not collected in the survey. In addition, given the design of the survey questionnaire, it was not possible to analyze the influence of the various symptoms and comorbidities of PsA on the treatment received or the patients’ perception of disease severity. Selection bias may have affected these analyses; patients who are satisfied with their treatment are less likely to respond to these types of questionnaires, so this type of survey may select for patients who are unhappy with their treatment. Only patients with PsA who completed the arthritis module of the NHWS and provided information on treatment were included in the analysis; 9% and 13% of US and EU5 respondents, respectively, with self-reported PsA were excluded from the analysis because of missing data. Data were limited to those obtained from patients who volunteered to participate, and therefore, results may not be applicable to the entire PsA population. Although this study included patients from the US and five European countries, it may not reflect clinical experience in all countries. Finally, although adherence was recorded, reasons for lack of adherence were not recorded, and it is possible that adherence was lower in patients who felt their treatments did not work or were too expensive. In addition, results here were not adjusted by age or gender, which may limit their interpretation.

Finally, although disease severity in patients receiving advanced and other therapies was reported to have changed after initiating treatment, 40–60% of patients reported moderate-to-severe PsA while being treated. The findings underscore the need for overall better management and identification of PsA, which, as demonstrated in this study, has a substantial impact on patients’ mental and physical health, employment, and healthcare resource utilization. Key areas for improvement include the early recognition of the disease and utilization of the guideline recommendation of a shared decision-making process between patient and physician on a treatment strategy to achieve remission or, alternatively, low disease activity as a treatment goal.

## Electronic supplementary material

Below is the link to the electronic supplementary material.

Supplementary material 1 (DOCX 53 KB)

Supplementary material 2 (DOCX 45 KB)

Supplementary material 3 (DOCX 45 KB)

Supplementary material 4 (DOCX 136 KB)
