# Supplementary material for: Treatment patterns, unmet need, and impact on patient-reported outcomes of psoriatic arthritis in the United States and Europe
Source: Rheumatol Int. 2018 Nov 13;39(1):121–30. doi: 10.1007/s00296-018-4195-x (PMC6329738; doi:10.1007/s00296-018-4195-x)
Supplement: Supplementary file 1 — Supplementary material 1 (DOCX 53 KB) [file 296_2018_4195_MOESM1_ESM.docx]

Online Resource 1

Treatment patterns, unmet need, and impact on patient-reported outcomes of psoriatic arthritis in the United States and Europe

**Journal:** *Rheumatology International*

Alice Gottlieb^1^ • Jordi Gratacos^2^ • Ara Dikranian^3^ • Astrid van Tubergen^4^ • Lara Fallon^5^ • Birol Emir^6^ • Laraine Aikman^7^ • Timothy Smith^6^ • Linda Chen^6^

*^1^Department of Dermatology, New York Medical College at Metropolitan Hospital, New York, NY, USA; ^2^Department of Rheumatology, University Hospìtal Parc Taulí Sabadell, Barcelona, Spain; ^3^Cabrillo Center for Rheumatic Disease, San Diego, CA, USA; ^4^Department of Medicine, Division of Rheumatology, Maastricht University Medical Center, Maastricht, Netherlands; ^5^Pfizer Canada, Montreal, QC, Canada; ^6^Pfizer Inc, New York, NY, USA; ^7^Pfizer Ltd, Sandwich, UK*

**🖂** Alice Gottlieb, Department of Dermatology, New York Medical College at Metropolitan Hospital, 1901 First Avenue, Floor 14B, New York, NY 10021, USA.
Tel: +1 (212) 423-7467. Fax: +1 (212) 423-8464. E-mail: [alicegottliebderm@gmail.com](mailto:alicegottliebderm@gmail.com)

**Online Resource 1.** Table showing demographics and characteristics of EU5 respondents who reported a diagnosis of PsA by country

|  | **France**  **Current treatment reported** | | | **Germany**  **Current treatment reported** | | | **Italy**  **Current treatment reported** | | | **Spain**  **Current treatment reported** | | | **UK**  **Current treatment reported** | | |
| --- | --- | --- | --- | --- | --- | --- | --- | --- | --- | --- | --- | --- | --- | --- | --- |
|  | **Advanced therapies**  **N = 9** | **Other therapies**  **N = 56** | **No current treatment**  **N = 116** | **Advanced therapies**  **N = 20** | **Other therapies**  **N = 79** | **No current treatment**  **N = 201** | **Advanced therapies**  **N = 16** | **Other therapies**  **N = 41** | **No current treatment**  **N = 117** | **Advanced therapies**  **N = 8** | **Other therapies**  **N = 20** | **No current treatment**  **N = 52** | **Advanced therapies**  **N = 16** | **Other therapies**  **N = 74** | **No current treatment**  **N = 122** |
| Age in years, mean (SD) | 45.4  (14.3) | 55.6 (16.5)^†††^ | 45.1 (16.1) | 54.7  (11.4) | 56.3 (12.2) | 54.7 (15.2) | 53.3  (12.3) | 56.6 (12.3)^††^ | 48.2 (15.7) | 52.3  (14.0) | 56.1 (13.7) | 49.4 (17.1) | 44.6 (13.3)*** | 57.5 (12.7)^†††^ | 47.2 (16.9) |
| Females, n (%) | 5  (55.6) | 38  (67.9) | 64  (55.2) | 9  (45.0) | 46  (58.2) | 105  (52.2) | 11  (68.8) | 21  (51.2) | 53  (45.3) | 4  (50.0) | 13  (65.0) | 23  (44.2) | 7  (43.8)* | 55  (74.3)^††^ | 66  (54.1) |
| White ethnicity, n (%) | NR | NR | NR | NR | NR | NR | NR | NR | NR | NR | NR | NR | NR | NR | NR |
| Employed, n (%)^a^  Employed full-time^b^  Employed part-time^b^  Self-employed^b^ | 4 (44.4)  2 (50.0)  2 (50.0)  0 | 19 (33.9)  13 (68.4)  4 (21.1)  2 (10.5) | 72 (62.1)  53 (73.6)  13 (18.1)  6 (8.3) | 11 (55.0)  7 (63.6)  3 (27.3)  1 (9.1) | 34 (43.0)  19 (55.9)  12 (35.3)  3 (8.8) | 97 (48.3)  57 (58.8)  26 (26.8)  14 (14.4) | 8 (50.0)  3 (37.5)  4 (50.0)  1 (12.5) | 20 (48.8)  10 (50.0)  3 (15.0)  7 (35.0) | 73 (62.4)  43 (58.9)  14 (19.2)  16 (21.9) | 5 (62.5)  4 (80.0)  0  1 (20.0) | 13 (65.0)  6 (46.2)  5 (38.5)  2 (15.4) | 34 (65.4)  21 (61.8)  7 (20.6)  6 (17.6) | 10 (62.5)  8 (80.0)  1 (10.0)  1 (10.0) | 25 (33.8)  17 (68.0)  3 (12.0)  5 (20.0) | 76 (62.3)  53 (69.7)  16 (21.1)  7 (9.2) |
| Health insurance, N, n (%)^c^ | 9 9  (100.0) | 54 54  (100.0) | 106 106  (91.5) | 20 20  (100.0) | 79 78  (98.7) | 201 195  (97.0) | 12 12  (100.0) | 39 39  (100.0) | 98 98  (100.0) | 8 8  (100.0) | 20 20  (100.0) | 50 50  (100.0) | 14 14  (100.0) | 59 59  (100.0) | 107 107  (100.0) |
| BMI kg/m^2^, n (%)  N  <18.5  18.5–<25  25–<30  ≥30 | 9  0  5 (55.6)  1 (11.1)  3 (33.3) | 55  5 (9.1)  15 (27.3)  22 (40.0)  13 (23.6) | 112  6 (5.4)  45 (40.2)  37 (33.0)  24 (21.4) | 20  0  1 (5.0)  6 (30.0)  13 (65.0) | 74  0  22 (29.7)  22 (29.7)  30 (40.5) | 194  3 (1.6)  59 (30.4)  71 (36.6)  61 (31.4) | 15  6 (40.0)  3 (20.0)  6 (40.0) | 39  0  13 (33.3)  14 (35.9)  12 (30.8) | 116  5 (4.3)  48 (41.4)  36 (31.0)  27 (23.3) | 8  0  3 (37.5)  4 (50.0)  1 (12.5) | 20  0  8 (40.0)  7 (35.0)  5 (25.0) | 51  4 (7.8)  19 (37.2)  20 (39.2)  8 (15.7) | 11  0  6 (54.6)  2 (18.2)  3 (27.3) | 65  2 (3.1)  14 (21.5)  23 (35.4)  26 (40.0) | 88  5 (5.7)  26 (30.0)  32 (36.4)  25 (28.4) |
| Current smoker, n (%) | 6 (66.7)**^†^ | 12  (21.4) | 35  (30.2) | 5  (25.0) | 22  (27.9) | 80  (39.8) | 7  (43.8) | 20  (48.8)^††^ | 30  (25.6) | 7  (87.5)*^†††^ | 7  (35.0) | 13  (25.0) | 8  (50.0) | 21  (28.4) | 37  (30.3) |
| Adjusted Charlson Comorbidity Index score, mean (SD)^d^ | 0.78  (1.64) | 0.64 (1.18) | 0.37 (0.89) | 0.90  (0.97) | 1.24 (1.51)^††^ | 0.74 (1.42) | 0.56  (1.09) | 0.46 (0.81) | 0.32 (0.74) | 1.88  (2.30) | 1.00 (1.38) | 0.42 (0.78) | 3.06  (5.92) | 0.59 (0.89) | 0.37 (1.02) |

**P* ≤ 0.05, ***P* < 0.01, ****P* < 0.001 vs. other; ^†^*P* ≤ 0.05, ^††^*P* ≤ 0.01, ^†††^*P* ≤ 0.001 vs. no treatment within each country

^a^Full- or part-time employment or self-employed
^b^Calculated as a proportion of total employed
^c^Health insurance not reported for EU5 as healthcare systems vary in European countries. Based on response (yes/no) to question ‘Do you currently have health insurance?’
^d^Higher scores represent greater comorbidity

*BMI* body mass index; *EU5* France, Germany, Italy, Spain, UK; *NA* not applicable; *NR* not recorded; *PsA* psoriatic arthritis; *SD* standard deviation
