# Supplementary material for: Treatment patterns, unmet need, and impact on patient-reported outcomes of psoriatic arthritis in the United States and Europe
Source: Rheumatol Int. 2018 Nov 13;39(1):121–30. doi: 10.1007/s00296-018-4195-x (PMC6329738; doi:10.1007/s00296-018-4195-x)
Supplement: Supplementary file 2 — Supplementary material 2 (DOCX 45 KB) [file 296_2018_4195_MOESM2_ESM.docx]

**Online Resource 2**

**Treatment patterns, unmet need, and impact on patient-reported outcomes of psoriatic arthritis in the United States and Europe**

**Journal:** *Rheumatology International*

Alice Gottlieb^1^ • Jordi Gratacos^2^ • Ara Dikranian^3^ • Astrid van Tubergen^4^ • Lara Fallon^5^ • Birol Emir^6^ • Laraine Aikman^7^ • Timothy Smith^6^ • Linda Chen^6^

*^1^Department of Dermatology, New York Medical College at Metropolitan Hospital, New York, NY, USA; ^2^Department of Rheumatology, University Hospìtal Parc Taulí Sabadell, Barcelona, Spain; ^3^Cabrillo Center for Rheumatic Disease, San Diego, CA, USA; ^4^Department of Medicine, Division of Rheumatology, Maastricht University Medical Center, Maastricht, Netherlands; ^5^Pfizer Canada, Montreal, QC, Canada; ^6^Pfizer Inc, New York, NY, USA; ^7^Pfizer Ltd, Sandwich, UK*

**🖂** Alice Gottlieb, Department of Dermatology, New York Medical College at Metropolitan Hospital, 1901 First Avenue, Floor 14B, New York, NY 10021, USA.
Tel: +1 (212) 423-7467. Fax: +1 (212) 423-8464. E-mail: [alicegottliebderm@gmail.com](mailto:alicegottliebderm@gmail.com)

**Online Resource 2.** Table showing types of US health insurance of survey respondents who reported a diagnosis of PsA

|  | **US patients**  **Current treatment reported** | | |
| --- | --- | --- | --- |
|  | **Advanced therapies**  **N = 225** | **Other therapies**  **N = 172** | **No current treatment**  **N = 640** |
| Health insurance, n (%) | 209 (93.0%)***^†††^ | 164 (95.3%) | 541 (84.5%) |
| Insurance coverage through an employer, n (%)  Current or former  Spouse's/partner's  Parent's or legal guardian's | 75 (35.9%)  26 (12.4%)  0 (0.0%) | 34 (20.7%)  20 (12.2%)  1 (0.6%) | 147 (27.2%)  67 (12.4%)  7 (1.3%) |
| Individual/Family insurance plan through a State Health Exchange, n (%) | 10 (4.8%) | 8 (4.9%) | 49 (9.1%) |
| Individual/Family insurance plan purchased directly by the patient, n (%) | 37 (17.7%) | 13 (7.9%) | 37 (6.8%) |
| Medicaid (MediCal for California residents) | 7 (3.4%) | 16 (9.8%) | 66 (12.2%) |
| Medicare | 51 (24.4%) | 62 (37.8%) | 150 (27.7%) |
| Veterans administration/CHAMPUS | 2 (1.0%) | 8 (4.9%) | 12 (2.2%) |
| TRICARE | 1 (0.5%) | 2 (1.2%) | 6 (1.1%) |

****P* < 0.001 vs. other treatment; ^†††^*P* < 0.001 vs. no treatment

*CHAMPUS* Civilian Health and Medical Program of the Uniformed Services; *PsA* psoriatic arthritis
