# Supplementary material for: Treatment patterns, unmet need, and impact on patient-reported outcomes of psoriatic arthritis in the United States and Europe
Source: Rheumatol Int. 2018 Nov 13;39(1):121–30. doi: 10.1007/s00296-018-4195-x (PMC6329738; doi:10.1007/s00296-018-4195-x)
Supplement: Supplementary file 3 — Supplementary material 3 (DOCX 45 KB) [file 296_2018_4195_MOESM3_ESM.docx]

**Online Resource 3**

**Treatment patterns, unmet need, and impact on patient-reported outcomes of psoriatic arthritis in the United States and Europe**

**Journal:** *Rheumatology International*

Alice Gottlieb^1^ • Jordi Gratacos^2^ • Ara Dikranian^3^ • Astrid van Tubergen^4^ • Lara Fallon^5^ • Birol Emir^6^ • Laraine Aikman^7^ • Timothy Smith^6^ • Linda Chen^6^

*^1^Department of Dermatology, New York Medical College at Metropolitan Hospital, New York, NY, USA; ^2^Department of Rheumatology, University Hospìtal Parc Taulí Sabadell, Barcelona, Spain; ^3^Cabrillo Center for Rheumatic Disease, San Diego, CA, USA; ^4^Department of Medicine, Division of Rheumatology, Maastricht University Medical Center, Maastricht, Netherlands; ^5^Pfizer Canada, Montreal, QC, Canada; ^6^Pfizer Inc, New York, NY, USA; ^7^Pfizer Ltd, Sandwich, UK*

**🖂** Alice Gottlieb, Department of Dermatology, New York Medical College at Metropolitan Hospital, 1901 First Avenue, Floor 14B, New York, NY 10021, USA.
Tel: +1 (212) 423-7467. Fax: +1 (212) 423-8464. E-mail: [alicegottliebderm@gmail.com](mailto:alicegottliebderm@gmail.com) **Running head:** Real-world treatment patterns in psoriatic arthritis

**Online Resource 3.** Table showing types of EU5 health insurance of survey respondents who reported a diagnosis of PsA

|  | **EU5 patients**  **Current treatment reported** | | |
| --- | --- | --- | --- |
|  | **Advanced therapies**  **N = 69** | **Other therapies**  **N = 270** | **No current treatment**  **N = 608** |
| Health insurance, n (%) | 63 (91.3%) | 251 (93.0%)^†^ | 556 (91.4%) |
| National Health Insurance/Public, n (%)^a^ | 34 (79.1%) | 132 (76.7%) | 247 (69.6%) |
| Private Health Insurance, n (%)^a^ | 6 (14.0%) | 22 (12.8%) | 78 (22.0%) |
| Mandatory Medical Coverage, n (%)^b^ | 3 (7.0%) | 18 (10.5%) | 30 (8.5%) |
| Public, without additional private insurance, n (%)^c^ | 12 (60.0%) | 51 (64.6%) | 128 (63.7%) |
| Public, along with additional private insurance, n (%)^c^ | 5 (25.0%) | 21 (26.6%) | 34 (16.9%) |
| Private insurance, n (%)^c^ | 1 (5.0%) | 4 (5.1%) | 21 (10.5%) |
| Aid entitlement and additional private insurance, n (%)^c^ | 2 (10.0%) | 2 (2.5%) | 11 (5.5%) |
| Other, n (%)^c^ | 0 (0.0%) | 0 (0.0%) | 1 (0.5%) |
| None of the above, n (%)^c^ | 0 (0.0%) | 1 (1.3%) | 6 (3.0%) |

^†^*P* < 0.05 vs. no treatment

^a^France, Italy, Spain, and UK

^b^France only

^c^Germany only

*EU5 F*rance, Germany, Italy, Spain, UK; *PsA* psoriatic arthritis
