# Supplementary material for: Treatment patterns, unmet need, and impact on patient-reported outcomes of psoriatic arthritis in the United States and Europe
Source: Rheumatol Int. 2018 Nov 13;39(1):121–30. doi: 10.1007/s00296-018-4195-x (PMC6329738; doi:10.1007/s00296-018-4195-x)
Supplement: Supplementary file 4 — Supplementary material 4 (DOCX 136 KB) [file 296_2018_4195_MOESM4_ESM.docx]

**Online Resource 4**

**Treatment patterns, unmet need, and impact on patient-reported outcomes of psoriatic arthritis in the United States and Europe**

**Journal:** *Rheumatology International*

Alice Gottlieb^1^ • Jordi Gratacos^2^ • Ara Dikranian^3^ • Astrid van Tubergen^4^ • Lara Fallon^5^ • Birol Emir^6^ • Laraine Aikman^7^ • Timothy Smith^6^ • Linda Chen^6^

*^1^Department of Dermatology, New York Medical College at Metropolitan Hospital, New York, NY, USA; ^2^Department of Rheumatology, University Hospìtal Parc Taulí Sabadell, Barcelona, Spain; ^3^Cabrillo Center for Rheumatic Disease, San Diego, CA, USA; ^4^Department of Medicine, Division of Rheumatology, Maastricht University Medical Center, Maastricht, Netherlands; ^5^Pfizer Canada, Montreal, QC, Canada; ^6^Pfizer Inc, New York, NY, USA; ^7^Pfizer Ltd, Sandwich, UK*

**🖂** Alice Gottlieb, Department of Dermatology, New York Medical College at Metropolitan Hospital, 1901 First Avenue, Floor 14B, New York, NY 10021, USA.
Tel: +1 (212) 423-7467. Fax: +1 (212) 423-8464. E-mail: [alicegottliebderm@gmail.com](mailto:alicegottliebderm@gmail.com)

**Online Resource 4.** Figure showing severity of PsA prior to and when receiving treatment based on patient self-report in individual EU5 countries


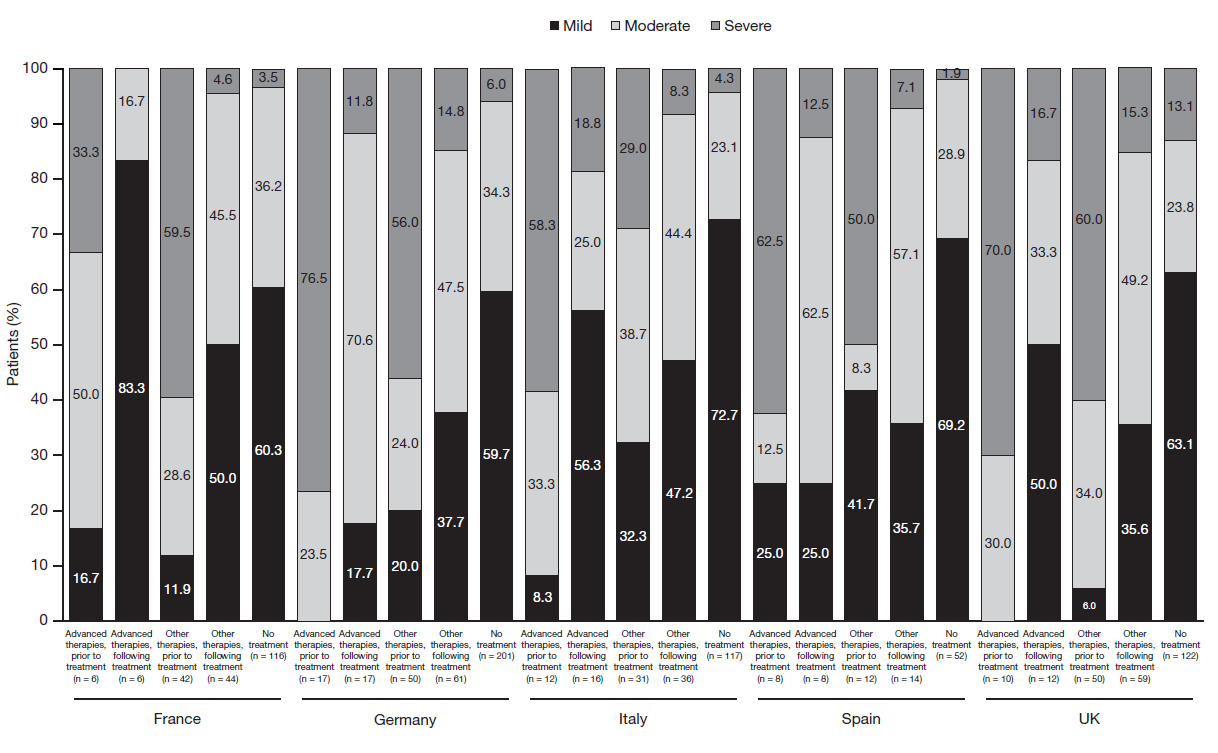


There were no statistically significant differences between treatments in any of the individual countries

*EU5* France, Germany, Italy, Spain, UK; *PsA* psoriatic arthritis
